# Relationships Among Types of Activity Engagement and Sleep Quality Among Older Adults

**DOI:** 10.1093/geroni/igaa057.1385

**Published:** 2020-12-16

**Authors:** Da Eun Kim, Tonya Roberts

**Affiliations:** University of Wisconsin–Madison, Madison, Wisconsin, United States

## Abstract

There is increasing awareness that lack of activity engagement is associated with poor sleep quality. However, the majority of studies have focused on the effect of a single type of activity engagement on sleep quality. Little is known about the combined effect of multiple types of activity engagement on sleep quality. The aim of this study is to identify relationships among different types of activity engagement and sleep quality among older adults. This study is a secondary data analysis using the Health and Retirement Study data. The participants included 3,357 persons who were age 65 or older and who responded to survey modules on activity engagement and sleep quality in 2016. Before we conducted primary analysis, factor analyses and calculating coefficient omega were conducted to identify factor structure, construct validity and reliability of the activity engagement questionnaire. Then, regression was conducted to examine the relationships among multiple types of activity engagement and sleep quality after adjusting for covariates based on the senescent sleep model. Exploratory and confirmatory factor analysis showed the 14-item questionnaire was comprised of three factors; social, cognitive, and physical activity and the three-factor model showed adequate validity and reliability. In the regression model social (β=0.25, p=0.033) and cognitive (β=0.36, p=0.001) activity engagement were positively related to better sleep quality. Based on these results, future research is needed to identify the mechanisms in which social and cognitive activities influence sleep quality positively and to develop targeted activity interventions for older adults.

